# MR Spectroscopy in Prostate Cancer: New Algorithms to Optimize Metabolite Quantification

**DOI:** 10.1371/journal.pone.0165730

**Published:** 2016-11-10

**Authors:** Giovanni Bellomo, Francesco Marcocci, David Bianchini, Emilio Mezzenga, Vincenzo D’Errico, Enrico Menghi, Romano Zannoli, Anna Sarnelli

**Affiliations:** 1 Medical Physics Unit, Istituto Scientifico Romagnolo per lo Studio e la Cura dei Tumori (IRST) IRCCS, Meldola, FC, Italy; 2 Experimental, Diagnostic and Specialty Medicine Department DIMES, University of Bologna, Bologna, Italy; Mayo Clinic Rochester, UNITED STATES

## Abstract

Prostate cancer (PCa) is the most common non-cutaneous cancer in male subjects and the second leading cause of cancer-related death in developed countries. The necessity of a non-invasive technique for the diagnosis of PCa in early stage has grown through years. Proton magnetic resonance spectroscopy (^1^H-MRS) and proton magnetic resonance spectroscopy imaging (^1^H-MRSI) are advanced magnetic resonance techniques that can mark the presence of metabolites such as citrate, choline, creatine and polyamines in a selected voxel, or in an array of voxels (in MRSI) inside prostatic tissue. Abundance or lack of these metabolites can discriminate between pathological and healthy tissue. Although the use of magnetic resonance spectroscopy (MRS) is well established in brain and liver with dedicated software for spectral analysis, quantification of metabolites in prostate can be very difficult to achieve, due to poor signal to noise ratio and strong *J-*coupling of the citrate. The aim of this work is to develop a software prototype for automatic quantification of citrate, choline and creatine in prostate. Its core is an original fitting routine that makes use of a fixed step gradient descent minimization algorithm (FSGD) and MRS simulations developed with the GAMMA libraries in C++. The accurate simulation of the citrate spin systems allows to predict the correct *J*-modulation under different NMR sequences and under different coupling parameters. The accuracy of the quantifications was tested on measurements performed on a Philips Ingenia 3T scanner using homemade phantoms. Some acquisitions in healthy volunteers have been also carried out to test the software performance *in vivo*.

## Introduction

Prostate cancer (PCa) is the most common cancer in men, and the second leading cause of cancer-related death in developed countries [1]. Traditionally, PCa detection and local staging depend on a combination of different tests, such as serum prostate-specific antigen (PSA) and digital rectal examination (DRE) [2,3]. Correct assessment of the tumor stage is crucial for disease management. *In vivo* magnetic resonance techniques (^1^H-MRS and ^1^H-MRSI) have been proved to be suitable noninvasive methods both to aid accurate detection of PCa [4,5] and to improve the definition of tumor extent [3] because metabolic alteration represents an hallmark of cancer [6]. High Resolution Magic Angle Spinning Magnetic Resonance Spectroscopy (HR-MAS) is a powerful tool that provides magnetic resonance spectra of *ex vivo* sample [6,7]. HR-MAS is an high field Nuclear Magnetic Resonance (NMR). It allows to obtain high resolution spectra, and for this reason many more metabolites are detectable with respect to *in vivo* ^1^H-MRS [6,7], providing a survey about PCa metabolomics. *Ex vivo* (HR-MAS) and *in vivo* (^1^H-MRS) studies show that metabolites, such as citrate (Cit), choline (Cho), creatine (Cr) and even polyamines are related with PCa [8–11], furthermore their concentrations have a good sensitivity and specificity for the diagnosis of PCa [12–15]. In particular the ratios between their concentrations, i.e. (Cho+Cr)/Cit or Cho/Cit, are the variables of greatest clinical interest because of their correlation with the Gleason score [16]. Although the use of ^1^H-MRS to study metabolic changes in brain and liver tumors is well established and supported by the use of recognized research software (jMRUI [17], LCMODEL [18], and Tarquin [19]), the same is not true for PCa. The absence of a dedicated software for the prostate MRS signal analysis makes quantification not well suited for clinical practice, although some commercial software (i.e. jMRUI [20] and LCMODEL [21]) provide features and tools for simulation that fit the purpose. The main problem in quantitative prostate MRS is citrate quantification. Cit spectrum shows a strong *J*-coupling modulation dependent on pulse sequence parameters, especially on the echo time (TE) [22]. In order to take account of the coupling and properly quantify citrate, the spectrum has to be simulated in the same condition of pulse sequences. In this work, simulations of spectra were performed with a C++ package that uses density matrix formalism [23] to calculate the evolution of the magnetization under different pulse sequences. The aim of this work is the development of a homemade software that allows the automatic analysis of the MRS signals based on spectra simulations.

## Materials and Methods

### Phantom measurements

In order to calibrate and assess homemade software quantification ability, six phantoms (250-ml polyethylene bottles) were prepared with the solutions reported in Table 1, following the method used by García-Martín et al. [21]. All of them were filled with 250 ml distilled water containing 155 mM NaCl, and prepared at pH = 7. Only one phantom (n°6 in Table 1) was prepared at pH = 5, to identify possible changes in Cit spectrum due to pH variability (this phantom was not included in the quantification test). HCl and NaOH were used to regulate the pH. All the pH measurements were performed with Cyberscan 500 (Eutech Instruments) pH meter. Some metabolites contain acid or basic chemical groups and so, in solution, pH value determines whereas hydrogen nuclei are associated or not with acid (or basic) groups, affecting proton chemical shift values and *J*-modulation [24]. Choline chloride (≥98%, C7527 Sigma-Aldrich, St. Louis, MO, USA), sodium citrate dehydrate (≥99%, W302600 Sigma-Aldrich, St. Louis, MO, USA) and creatine monohydrate (≥98%, C3630 Sigma-Aldrich, St. Louis, MO, USA), were used respectively as Cho, Cit and Cr source. Concentrations of metabolites, NaCl and pH in phantoms were chosen also considering the work of Kavanagh et al. [25] to resemble physiological values. Paramagnetic salts were not used in phantoms. MR spectroscopy acquisitions were performed on a 3T MR scanner (Ingenia, Philips Healthcare, Best, The Netherlands) equipped with “Whole Body Transmitter Coil” incorporated into the bore (no external transmitter coils). “Ingenia Philips 3T Scanner Receiver Head Coil” (30 cm coverage, 15 channels, direct digital data sampling) was used for all the phantom measurements. Single voxel spectra (SVS) relative to phantom data were collected by means of Point Resolved Spectroscopy Sequences (PRESS) with three Chemical Shift Selective (CHESS) pulses for water suppression and a relatively long repetition time (TR). PRESS parameters were: TR/TE = 3000/140 ms, voxel size of 2×2×2.5 cm^3^ (positioned in the center of the phantoms), 1.2 cm slice thickness, 1024 points in resolution, 2000 Hz bandwidth (512 ms acquisition time), and 192 averages. PRESS was chosen because it has a better Signal to Noise Ratio (SNR) than the Stimulated Echo Acquisition Mode sequence (STEAM) [26].

**Table 1:** Composition of the homemade phantoms.

*n°*	*Cit (mM)*	*Cho (mM)*	*Cr (mM)*	*NaCl (mM)*	*pH*
*1*	5.0 ± 0.1	20.0 ± 0.2	16.1 ± 0.2	155 ± 1	7.00 ± 0.05
*2*	15.0 ± 0.1	15.0 ± 0.2	12.1 ± 0.2	155 ± 1	7.00 ± 0.05
*3*	25.0 ± 0.2	10.0 ± 0.2	9.4 ± 0.2	155 ± 1	7.00 ± 0.05
*4*	40.0 ± 0.3	7.5 ± 0.2	7.5 ± 0.1	155 ± 1	7.00 ± 0.05
*5*	60.0 ± 0.5	5.0 ± 0.1	5.4 ± 0.1	155 ± 1	7.00 ± 0.05
*6*	60.0 ± 0.5	0	0	155 ± 1	5.00 ± 0.05

### *In vivo* measurements

In order to test the fitting ability of the homemade software, *in vivo* spectra were acquired from two healthy volunteers. The volunteers signed appropriate informed consent and the study was approved by the institute ethic committee (“Comitato Etico IRST IRCCS-AVR”). The hardware was the same as for phantom acquisitions with the exception of the receiver coil, which was an Anterior Phased Array Coil (PAC) having 100 cm coverage, 52 channels and direct digital data sampling. From the first healthy volunteer, a single-voxel scan and a 3D PRESS (^1^H-MRSI) with 1024 points in resolution were acquired, while from the second volunteer only a 3D PRESS with 2048 points was acquired. In both cases, a 2000 Hz bandwidth (512 ms acquisition time) was used. The sequences were launched with TE = 140 ms (to see peaks of Cit approximately in-phase) and TR = 1500 ms. For ethical reasons, volunteers were not subjected to the standard preparation protocol. In particular, anticholinergics were not administered to diminish rectum movements. Number of averaged signals was reduced from 192 to 32 to achieve a shorter exam time (at the expense of SNR), while Specific Absorption Rate (SAR) and Peripheral Nerve Stimulation (PNS) modes were set to “low”. For the two healthy volunteers, five slices of 1.2 cm in thickness were acquired; in each slice there was a matrix of 9×7 voxels of 1×1×1.2 cm^3^ of volume (for a total of 315 spectra). Voxel size in single-voxel scan was 1.5×1.5×1.5 cm^3^ and the voxel was placed in the peripheral zone of the prostate gland. In the single-voxel spectrum acquisition three CHESS pulses were used for water suppression, while for 3D PRESS spectra acquisitions dual bandwidth Basing Pulse [27] to water and fat suppression were used instead.

### Software and data processing

**Fig 1:**
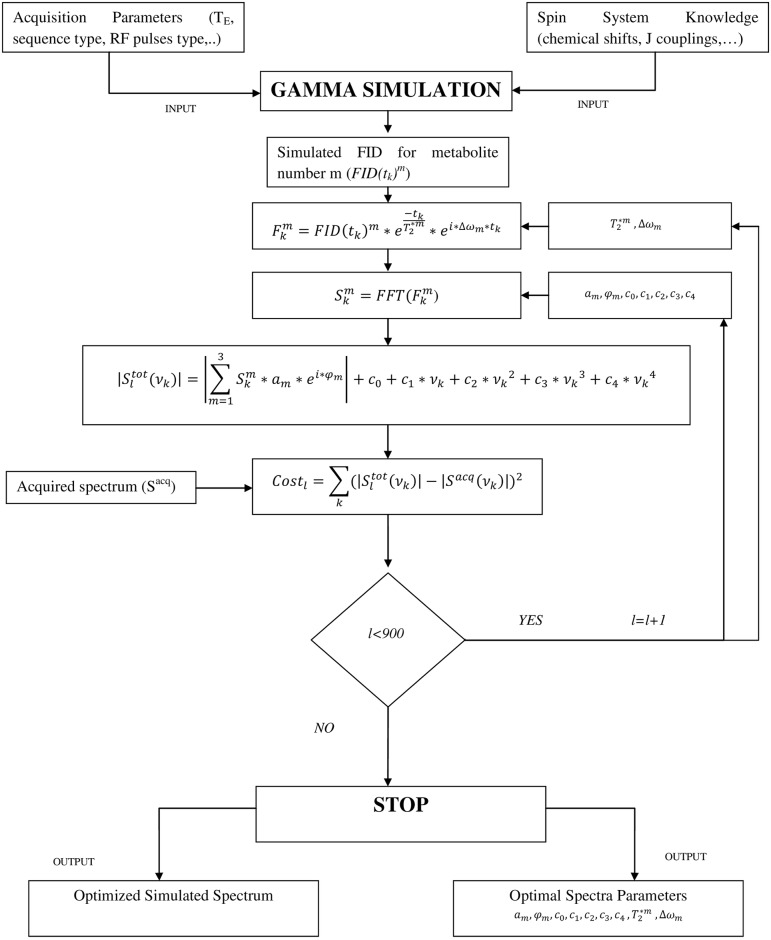
Block diagram of the homemade software for MRS signal analysis.

Spectroscopic data were analyzed by means of a homemade software, based on GAMMA C++ libraries [28] and on a gradient descent algorithm [29], running on Linux operating system. To avoid the divergence of the gradient during the iterative process, a fixed step approach like the one used in digital signal processing (FSGD) [30] was used. It allows the parameters to change only by fixed quantities, following the sign of the gradient but ignoring its magnitude. Before the iterative fitting process starts, all the *in vivo* spectra are smoothed with an automatic apodization of 2 Hz. No smoothing was used for phantoms, since the SNR was already acceptable. A detailed explanation of the principal functions used by the software is provided in the Appendix, while a block diagram of the software algorithm is shown in Fig 1.

GAMMA package for MRS simulations is based on density matrix formalism to make previsions about NMR spectra after a RF pulse sequence is applied. Density matrix formalism is an useful tool to describe the behavior of strong-coupled spins systems, such as the hydrogenous nuclei in the two equivalent methylene groups of Cit [21, 23]. In order to simulate the spin systems, the software requires a text file (“Spin system knowledge” block in Fig 1) containing the NMR properties of each metabolite (i.e. the isotope type, frequencies for each proton in the metabolite, the scalar coupling between each proton couple and the spectrometer frequency). All the NMR parameters were measured from the spectra both for *in vitro* and *in vivo* quantifications (see the Appendix for more details). The acquisition parameters used for the specific NMR sequence (i.e. TE in pulse sequence, sampling time and resolution) are given as input through a graphical user interface. The free induction decay (FID) signal of an ideal PRESS sequence is simulated for each metabolite by means of the GAMMA methods. The software Fourier transforms the simulated FIDs and computes the weighted sum of the metabolites simulated spectra using an appropriate model and initial set of parameters (see appendix). Afterwards, the software compares the model spectrum to the measured one using a cost function. Finally, it updates the parameters and proceeds to the next iteration minimizing the cost function. The iterative process takes a defined number of steps (900 steps were selected for the purpose). When the iterative process terminates, the software gives back the optimized simulated spectrum together with the output parameters of the simulated metabolites (amplitude, relative frequency, relative phase, etc.). Since the software is able to define the spectrum of each metabolite (see appendix and Fig 1), it is possible calculate the area (or integral) of each metabolite. Areas are calculated by summing the simulated points of the metabolite spectra on the whole frequency interval (i.e. the selected 2000 Hz in bandwidth). For comparison, spectra quantifications were performed also using AMARES algorithm (in jMRUI software). AMARES deconvolves a sampled FID in a series of exponentially damped sinusoids [31]. AMARES peak parameters were determined using the “peak picking” function with no prior knowledge. Furthermore, jMRUI software provides also a package, (named QUEST [32]) able to quantify spectra using simulated FIDs. The simulated basis set can be built using NMRscopeB [33], a simulation tool included in the jMRUI package. QUEST algorithm was used in the comparative analysis too. The metabolite basis set was created using NMRscopeB, Cit was modeled with the same parameters used for the homemade software analysis, while chemical shift and multiplicities of Cho and Cr where chosen to produce single peaks at frequencies of 3.27 ppm (Cho) and two peaks at 3.0 and 3.9 ppm (Cr).

### Statistical analysis

In order to assess the goodness of the fit, the Kolmogorov-Smirnov test (K-S Test) [29] was used in every quantification, comparing the simulated spectrum with the acquired one in the interval comprised between 2.1 and 3.6 ppm, which is the one where the principal peaks of the metabolites can be identified. K-S test was performed using the Matlab function “kstest2”. This function takes as input the simulated and measured spectra, and returns a test decision for the null hypothesis (i.e. the spectra come from the same continuous distribution), giving p and D values of K-S test. In this test, the D value is the maximum difference between the cumulative distributions (integral of the spectra) and p-value is the probability that to reject the null hypothesis is a mistake. If p value is high (greater than 0.05) there is not enough evidence to reject the null hypothesis. Metabolite quantifications were also performed using jMRUI software [17], in order to test the performances of the homemade software in comparison to a commercial one. The agreement of the metabolites ratios, calculated using both homemade software and AMARES [31] algorithm (in jMRUI), in comparison to the real concentration ratios was estimated in terms of r^2^ coefficients. Finally K-S test was made also to compare AMARES calculated spectra and the measured ones in the same interval (i.e. 2.1–3.6 ppm).

## Results

**Fig 2:**
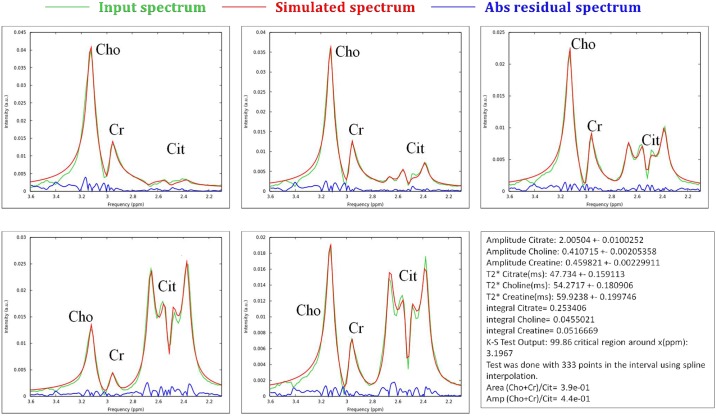
Comparison between acquired smoothed (green lines) and simulated (red lines) spectra, together with their absolute difference (blue lines), for the phantoms considered in the study and reported in Table 1: phantom a) n°1, b) n°2, c) n°3, d) n°4 and e) n°5. f) An example of the output file of the homemade software with the final simulation parameters obtained for phantom n°4.

Fig 2a–2e shows a comparison between the acquired and the simulated spectra for all phantoms considered in this study. The horizontal axis reports the frequency interval where only metabolite peaks are visible and no water residual signal is present. In the same plots, the absolute difference between the two spectra (measured and simulated) is also shown. In Table 2 are summarized the results of the K-S test for the AMARES and the homemade software. At first instance, the homemade software reproduced the metabolites spectra correctly in each phantom: the identity between the measured and simulated spectra is not refutable by K-S test. However, it is refutable in all phantom quantification except the number 2 for the AMARES algorithm.

**Table 2:** Kolmogorov-Smirnov analysis in phantom spectra using AMARES and homemade software. The number referring the phantom is the same of Table 1.

*AMARES*			*homemade software*		
n°	p-value	D-value	n°	p-value	D-value
1	0.0494	0.1786	1	0.9975	0.0602
2	0.4149	0.1161	2	0.9975	0.0602
3	0.015	0.2054	3	0.9972	0.0602
4	0.015	0.2054	4	0.9973	0.0602
5	0.015	0.2054	5	0.9975	0.0602

**Fig 3:**
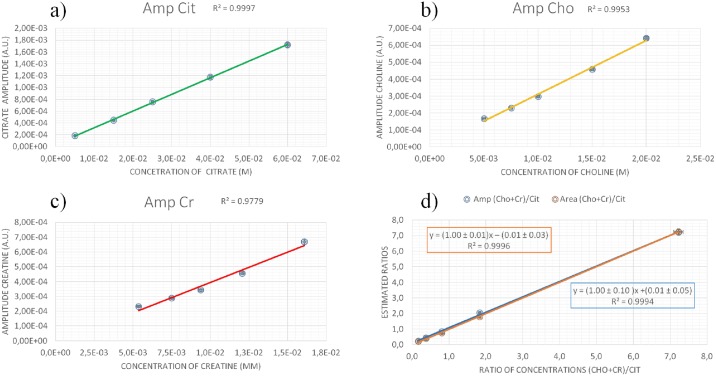
Results of the homemade software relative to the metabolite spectra amplitudes versus their concentrations for a) Cit, b) Cho and c) Cr in phantoms. d) (Cho+Cr)/Cit estimated ratios versus real concentration ratios in phantoms.

In phantoms, we found a good linear positive correlation between amplitudes (or area) of the metabolites estimated by software, and the real metabolite concentration (Fig 3a–3c). For example, the linear correlation between Cit amplitude vs Cit concentration has r^2^ coefficient of 0.9997 using the homemade software (Fig 3a). An excellent linear correlation between (Cho+Cr)/Cit concentration ratios and the same ratios estimated by software (Fig 3d) confirms the accuracy of the quantification, both using metabolite amplitude ratios and metabolite area ratios. Table 3 summarizes the quantification results showing (Cho+Cr)/Cit ratios estimated by homemade software in comparison to the real concentration ratios. Uncertainties on the estimated ratios are only lower bounds of the real uncertainties and are produced mainly from measuring T_2_ decay constants (see appendix for more information), while uncertainties in real concentration ratios are due to precision of the balance with which metabolites were weighed.

**Table 3:** (Cho+Cr)/Cit values estimated by homemade software (Amplitude and Area) are compared with the (Cho+Cr)/Cit values estimated by AMARES (jMRUI software) and the same real metabolites ratios inside phantoms.

*Concentration*	*Amplitude*	*Area*	*Amplitude (AMARES)*
7.2 ± 0.1	7.2 ± 0.2	7.2 ± 0.2	11.2 ± 0.3
1.83 ± 0.02	2.04 ± 0.05	1.77 ± 0.05	5.1 ± 0.1
0.80 ± 0.01	0.85 ± 0.03	0.76 ± 0.03	2.1 ± 0.1
0.375 ± 0.006	0.44 ± 0.03	0.39 ± 0.03	1.08 ± 0.03
0.167 ± 0.004	0.23 ± 0.03	0.20 ± 0.03	0.52 ± 0.01

**Fig 4:**
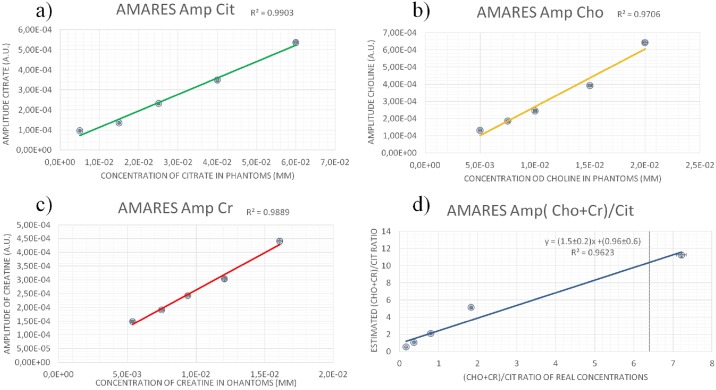
Results of the AMARES (jMRUI software) relative to the metabolite spectra amplitudes versus their concentrations for a) Cit, b) Cho and c) Cr in phantoms. d) (Cho+Cr)/Cit estimated ratios versus real ratio concentrations in phantoms.

In Fig 4, the results of metabolite amplitudes versus metabolite phantom concentrations obtained by means of the AMARES algorithm are shown, and in Table 3 the amplitude ratios estimated by AMARES software together the concentration ratios (Cho+Cr)/Cit are summarized. Even if AMARES algorithm gave a good linear relationship between the metabolite amplitudes and their concentrations, comparable to the ones obtained by the homemade software (Fig 4), it provides (Fig 4d) poor agreement with the real concentration ratios of metabolites inside the phantoms compared to the homemade software (Fig 3d and Table 3). QUEST routine was also tested but the overall results were meaningless, that is the algorithm was not able to fit some of the metabolites inside phantoms in every quantification: in spectra with “high” Cit concentration (e.g. phantom n°5), QUEST did not measure Cho and Cr, while in the other ones it was not able to measure Cit. The quantifications were repeated several times varying the simulation parameters, but the results were the same. Thus, QUEST analysis results are not shown here.

**Fig 5:**
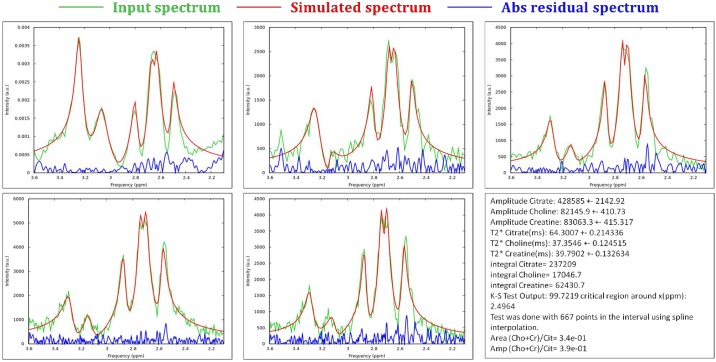
*In vivo* spectra comparison between acquired smoothed (green lines) and homemade software simulated (red lines) spectra, together with their absolute difference (blue lines). a) Single voxel spectrum of the peripheral zone of the prostate (Voxel size: 1.5⨯1.5⨯1.5 cm^3^, 1024 pts in resolution), b-c) two spectra taken from 3-D PRESS of subject 1 with 1024 pts in resolution, d-e) two spectra taken from 3-D PRESS of subject 2 with 2048 pts in resolution, f) output of the quantification of the spectrum d).

In Fig 5 *in vivo* spectra of the two volunteers are shown. Only a small apodization of 2 Hz was performed on the acquired signals.

## Discussion

The main aim of this work was to propose a new strategy to quantify prostate metabolites by MRS to improve the power of non-invasive diagnostics for PCa. The motivation that brought to the development of this tool was the absence of a dedicated routine for metabolite quantification in prostate especially for Cit. Indeed Cit has a strong *J*-modulation that makes its spectrum shape dependent on pulse sequences parameters and environmental conditions. This purpose was obtained by performing a rigorous quantum mechanical simulation of the hydrogen nuclei in the two equivalent methylene groups of Cit using the GAMMA C++ libraries.

The next problem was to find an appropriate cost function and a robust minimization algorithm. As it is shown in the appendix, the sum on the frequency space of the squared differences between the absolute values of the simulated and acquired spectrum was chosen as a cost function. The absolute value allows to ignore the global phase of the spectrum (while the relative phases are still considered) and makes the optimization less sensitive to local minima. FSGD was used to minimize the cost function, since it provides a simple minimization tool that ensures the convergence of the iteration process. The homemade software was firstly tested on phantoms made in our institute. Some studies have been performed to understand the biochemical environment of the prostate using ultra high field NMR spectroscopy [34]; these results can guide the experimenter in building up phantoms with chemical composition similar to human prostate. Because the goal of the study was only to test the homemade software, simple phantoms with only Cho, Cit and Cr were used, controlling the pH by addition of diluted HCl or NaOH solutions. Control of the pH in phantom is essential because chemical shifts and thus *J*-modulations are pH dependent. Significant differences in the coupling constant of Cit were not observed by changing the pH of the solution (from pH 7 to pH 5); conversely, different line-shapes and different values of chemical shifts of Cit protons were observed. An estimation in phantoms of chemical shifts and *J*-coupling value of Cit was done by measuring the differences between frequencies of peaks in Cit spectrum, using a short TE STEAM sequence (see appendix).

Because of the uncertainties of the estimated parameters (due to the resolution and field uniformity of a clinical MR scanner), the choice of *J*-coupling constants and Larmor frequencies for simulations was not univocal. PRESS sequences with long TR using different TE were used to measure (in phantom) the relaxation time (T_2_^m^) of the metabolites. Homemade software and jMRUI were used to measure metabolites amplitude (and spectral area) as function of TE to derive (from exponential fit) the T_2_^m^ relaxation values. These were measured to determine the correction factors required to compensate the transverse relaxation processes in metabolite quantifications (see appendix).

Consequently, the software ability to quantify the MR spectra was tested. A strong correlation between metabolite concentrations and amplitudes (or areas) estimated by homemade software was found, confirming the reliability of the model used to represent the molecules. The goodness of the quantifications was certainly due to the correct modeling of Cit spectrum, achieved by taking into account the *J*-modulation during the simulations with GAMMA. An analogue analysis was performed using a commercial software (AMARES algorithm of jMRUI software) but weaker correlations were found (in terms of r^2^ coefficients), and the estimated (Cho+Cr)/Cit ratios were not in agreement with the real ratio calculated by molar concentrations in the phantoms. This happens because metabolite peak selection performed by AMARES is quite ill-posed for multiplets like Cit, since AMARES doesn’t take into account *J-*modulation.

K-S test analysis was used both for the spectra calculated by homemade software and for the ones calculated by AMARES. For the homemade software, p-values obtained through the K-S test are always much higher than the critical level (0.05), implying that the null hypothesis can never be rejected. Conversely using AMARES in all the quantifications (except for phantom n°2), the null hypothesis can always be rejected. In scientific literature, LCMODEL is also used for quantitative MRS in the prostate [15, 21]. Unfortunately, the prostate basis set for LCMODEL is only available to the developer of this software and for an exclusive group of research centers. However, our software provided performances comparable to the ones obtained by using LCMODEL. Indeed it resulted that the correlation between amplitude of Cit vs Cit concentration in phantoms had r^2^ coefficient of 0.9997 using the homemade software, and of 0.986 in Garcia-Martin paper [21], in which LCMODEL was used for the quantification.

The last part of this work concerned *in vivo* tests on healthy volunteers. *In vivo* acquisitions were performed to test the ability of our algorithms to fit spectra in low SNR condition inside the prostatic tissue. The presence of ions and the different magnetic susceptibility between phantom and tissue could change dramatically the shape of the spectra, particularly Cit spectrum. This is because *J*-couplings and chemical shifts strongly depend on cations concentrations and pH [24] (for example, cations like Zn^++^ or Ca^++^ were not present in phantoms and also pH could be different between phantoms and prostate). Hence, a test about the ability of the software to fit *in vivo* spectra had to be made. Two 3D PRESS and a single-voxel scan were acquired from two subjects. At first, an estimation of the spectral parameters was performed as in phantom tests, to find a range of input simulation values for *in vivo* Cit (*J*-coupling and chemical shifts). Subsequently, the fitting ability of the homemade software was tested on common prostate spectra with low SNR. It was found that the routine was able to fit the line-shapes of Cit (given the right NMR parameters) also for *in vivo* conditions.

Although *in vivo* quantification was not the main purpose of this work (at least in this early stage), in Fig 5 of the manuscript there is the output file relative to a quantification performed in the central zone of the prostate of subject 2. The values found for the ratio (Cho+Cr)/Cit were 0.333 with amplitudes and 0.382 with integrals. Basharat et al. [20] obtained 0.4125 in the central zone for the same ratio. Although the values obtained in this paper and in Basharat look similar, it is not possible to affirm that they are in agreement because a more accurate error handling should be made. Furthermore the prostate concentration ratio in healthy subject has a statistical variance that cannot deduced by limited cohort of subjects used for *in vivo* tests.

The measurements for this work were performed on the Philips Ingenia 3T using phased array coils (PAC); an interesting issue is the comparison between PAC and endorectal coil (ERC) performances. Several papers have been published that compare receiver PAC with the more invasive ERC [16, 35,36]. Particularly, the use of ERC significantly improves spectral line width; however, good performances are obtained using PAC too. Therefore, the use of the external PAC may be recommended for patients with rectal diseases or patients who could not tolerate the discomfort of ERC insertion. ROC curve for cancer diagnosis based on MRS, obtained by using ERC, has an AUC (area under curve) very similar to the one obtained by using PAC (0.869 ERC vs 0.859 PAC) [35].

## Conclusion

Quantification of the citrate is one of the main problems concerning prostate MRS. The use of a structural NMR tool like GAMMA, associated with an optimization routine for amplitudes, frequency shifts, relative phases and effective decay constants allows to get reliable metabolite quantifications in prostatic tissue. In general, this approach could work well with all kinds of NMR spectra in metabolomics. The first results relative to our quantification routine are certainly encouraging but further tests are required to assess if this kind of strategy could help the non-invasive diagnosis of PCa at an early stage. The homemade software developed in this study produced better results (in terms of quantification and fitting capabilities) compared to the commercial one for all the phantom measurements. Furthermore, it showed good fitting capabilities *in vivo*, also in low SNR conditions. To improve the accuracy of *in vivo* measurements, our future purposes are mainly focused on considering polyamines quantification. Polyamines spectra commonly overlap to creatine and choline ones, thus creating errors and misunderstandings in MRS quantifications. Certainly to insert polyamines in the analysis will provide an improvement in prostate MRS spectra quantification.

## Appendix

### Algorithm description

A block diagram of the homemade software is shown in Fig 1. The software requires the NMR properties of the significant hydrogen groups of each metabolite (i.e. isotope type, metabolite proton frequencies, their multiplicity, scalar coupling between each proton couple and spectrometer frequency) as input, together with the NMR sequence parameters (i.e. TE, bandwidth and spectral resolution of the PRESS sequence used for acquisition). Using these input parameters, the software simulates Free Induction Decay (FID) signals FID(t_k_)^m^ of each metabolite (one molecule) at a sampled time point t_k_, being m the metabolite index. FIDs are shifted in frequency by an oscillating function e^iΔωmtk^ (Δω_m_ is the frequency shift of the m^th^ metabolite) and multiplied by a damped exponential in time-domain, in order to take into account the metabolite effective transverse relaxation (T_2_*^m^). Hence, the simulated signal can be written as:

$$F_{k}^{m}=FID{\left(t_{k}\right)}^{m}e^{-\frac{t_{k}}{T_{2}^{*m}}}e^{i\Delta \omega_{m}t_{k}}$$ (A1)

Successively, the complex spectrum of a single metabolite is obtained through Fast Fourier Transform (FFT) of its simulated signal Eq (A1):

$$S_{k}^{m}=FFT\left(F_{k}^{m}\right)$$ (A2)

The absolute value of the simulated spectrum of all the metabolites is computed as:

$$\left|S_{l}^{tot}\left(\nu_{k}\right)\right|=\left|\overset{}{\underset{}{\sum_{m=1}^{3}S_{k}^{m}a_{m}e^{i\varphi_{m}}}}\right|+c_{0}+c_{1}\nu_{k}+c_{2}\nu_{k}^{2}+c_{3}\nu_{k}^{3}+c_{4}\nu_{k}^{4}$$ (A3)

Here ν_k_ is the sampled spectrum frequency, *φ*_*m*_ its relative phase, the *c*_0_…*c*_4_ are the coefficients of the polynomial baseline and *a*_*m*_ is the amplitude of the metabolite signal. The subscript *l* stands for the current iteration number in the computation process. Starting parameters (i.e. amplitude, phase, etc.) for the simulation are obtained at the beginning of every quantification by a rough grid search performed by the program.

Once the simulated spectrum is computed, the software compares it iteratively with the acquired one, minimizing the cost function expressed as:

$${\text{ Cost}}_{l}=\sum_{k}{\left(\left|S_{l}^{tot}\left(\nu_{k}\right)\right|-\left|S^{acq}\left(\nu_{k}\right)\right|\right)}^{2}$$ (A4)

It considers the sum of the squared difference between the absolute values of the two spectra over the selected frequency interval. Spline interpolation was used to avoid binning errors in the computation of the cost function: the differences between the two spectra were evaluated by oversampling them on a number of points set as:

$$3\left(\nu_{k,Max}-\nu_{k,min}\right)/R$$ (A5)

where *v*_*k*,*Max*_ and *v*_*k*,*min*_ define the maximum and minimum frequency of the considered frequency interval, and *R* is the resolution used during MRS acquisition. At each iteration, the software updates the input parameters (i.e. *a*_*m*_, *φ*_*m*_, *c*_0_ … *c*_4_, Δω_m_ and T_2_^m^*) and recalculates the total simulated spectrum using Eq (A3). After 900 iterations the optimization is stopped. The optimized simulated spectrum, its parameters (*a*_*m*_, *φ*_*m*_, *c*_0_ … *c*_4_, Δω_m_ and and T_2_^m^*), and a graphical comparison between the simulated and acquired spectra (together with their absolute difference) are given as output results.

During the iteration process, the cost function is minimized by using a fixed step gradient descent algorithm (FSGD). This algorithm belongs to the family of Gradient descent methods (GD), a common iterative minimization approach which uses the gradient of a cost function to move across the parameter space to reach a minimum. It is a common choice to search minima inside parameter spaces with several dimensions. Generally, FSGD algorithm updates the parameters vector using the normalized gradient of the cost function in order to avoid divergences:

$$param_{l+1}\left(k\right)=param_{l}\left(k\right)-\frac{G{\left(k\right)}_{l}}{\left|G{\left(k\right)}_{l}\right|}dparam_{l}\left(k\right)$$ (A6)

*param*_*l*+1_ is the vector containing the parameters at iteration *l*+1 where the parameters are: *a*_*m*_, *φ*_*m*_, *c*_0_ … *c*_4_, Δω_m_ and T_2_*^m^ (m = 1,…,3). Besides *param*_*l*_(*k*) is the k^th^ component of the vector *param*_*l*_ at iteration *l*. ***G***_***l***_ is the cost function gradient at iteration *l* and *G*(*k*)_*l*_ is its k^th^ component.

$$G_{l}=\left(\frac{\partial cost_{l}}{\partial a_{m}},\;\frac{\partial cost_{l}}{\partial \varphi_{m}},\frac{\partial cost_{l}}{\partial \Delta \omega_{m}},\frac{\partial cost_{l}}{\partial T_{2}^{*m}},\ldots \right)\;m=1,\ldots 3$$ (A7)

In this approach, only the sign of the k^th^ component of the gradient is used, while the step of the descent is “preconditioned” by the step vector *dparam*_*l*_. Components of the step vector were chosen empirically and some of them change their values during the iteration. For example, step parameters related to polynomial baseline are constant and are chosen to produce very weak changes in the spectrum. Phase and shift step parameters are static too, while the ones related to $T_{2}^{*}$ decays and amplitudes of metabolites are updated at each iteration, since they are set to be a fixed ratio of their corresponding parameters. For example, the step vector for amplitude is chosen to be a 0.5% of the value found after the grid search and it is rescaled at each iteration:

$$da_{l+1}=\frac{a_{l}}{200}$$ (A8)

At the end of optimization, the amplitudes (and the integrals) are corrected in order to take account of different metabolite T_2_ values:

$$a_{m}^{corr}{}_{\;}=\frac{a_{m}*e^{\frac{TE}{T_{2}^{m}}}}{\frac{a_{w}}{2}*e^{\frac{TE}{T_{2}^{w}}}}$$ (A9)

Where TE is the sequence echo time, T_2_^m^ the transverse relaxation time of the metabolite m, *a*_*w*_ the amplitude of the water signal, T_2_^w^ the water relaxation time. $a_{m}^{corr}$ are the amplitudes comparable to concentrations (see Materials and Methods, Results, Tables 1–3, Figs 2–3). Amplitude of water signals was obtained from spectra without water suppression. This correction was performed also during the AMARES quantification.

### Measuring NMR parameters

**Fig 6:**
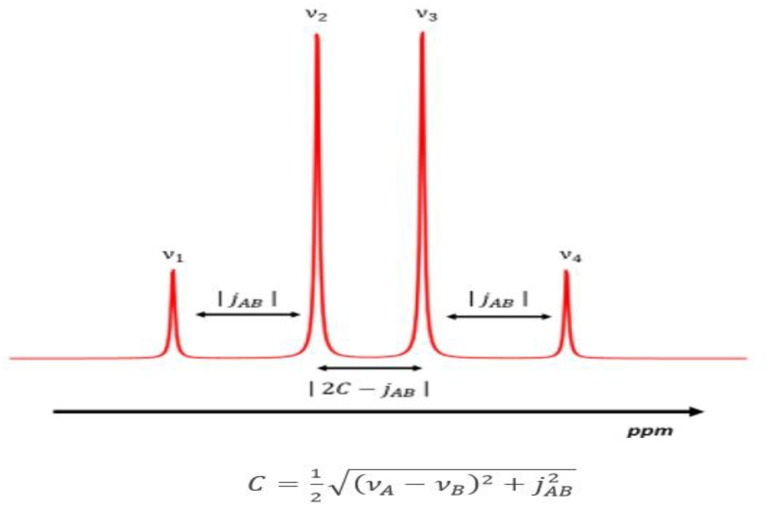
Methylene protons of Cit *are a strong-coupled spin system*.

Using the exact chemical shift value is not crucial for the quantification because the software is able to correct small deviations of metabolite chemical shift iteratively Eq (A1). A STEAM sequence with short echo time (8.6 ms) was used to measure initial Cit chemical shifts and Cit *J*-coupling in phantoms. Methylene protons of Cit are representable as a strong-coupled AB system (a typical AB system is depicted in Fig 6). This strong coupling model was used to estimate the *J*-coupling constant [37]:

$$J_{AB}=\frac{\left(\nu_{1}-\nu_{2}\right)+\left(\nu_{2}-\nu_{4}\right)}{2}$$ (A10)

In Eq (A10) ν_1,_ ν_2,_ ν_3_ and ν_4_ are the peak frequencies of Cit quartet. Furthermore, ν_A_ and ν_B_ are the frequencies of the Cit hydrogen nuclei. These frequencies can be calculated using the equations [37]:

$$\delta =\frac{\nu_{1}+\nu_{2}+\nu_{3}+\nu_{4}}{4};\;\;\;\;\;\;\Delta =\sqrt{{\left(\nu_{2}-\nu_{3}+j_{AB}\right)}^{2}-j_{AB}{}^{2}}$$ (A11)

and finally:

$$\nu_{A}=\frac{\Delta +2\delta }{2}\;;\;\;\;\nu_{B}=\frac{2\delta -\Delta }{2}$$ (A12)

Within the experimental uncertainties in the simulation ν_A_ = 2.44 ppm, ν_B_ = 2.56 ppm and J_AB_ = 15 Hz were set as input spectral parameters for Cit (results are in S1 Table). These values were taken by García-Martín et al. [21], but they are almost equal to the calculated ones (ν_A_ = 2.44 ppm, ν_B_ = 2.57, and J = 16 Hz). To obtain a faster execution, Cho methyl proton was simulated as a single peak centered at 3.12 ppm while Cr methyl resonance was set to 2.95 ppm (secondary peaks are ignored, while amplitude and area values are rescaled at the end of the iteration process for their real multiplicity). Due to the poor SNR of STEAM, *In vivo* parameters of Cit were estimated using a PRESS sequence with an echo time of 140 ms, chemical shifts and *J-*coupling were then measured with the method used in phantoms (S2 Table).

Although the uncertainties are quite big, due to the poor SNR, the values in S2 Table are agreement with the results of García-Martín et al. [21]. For the simulations we used ν_A_ = 2.602 ppm, ν_B_ = 2.708 ppm and J_AB_ = 16.8 Hz for Cit. Cho methyl proton was simulated as a single peak centered at 3.29 ppm while Cr methyl resonance was set to 3.1 ppm.

#### Measuring T_2_ decay constants

**Fig 7:**
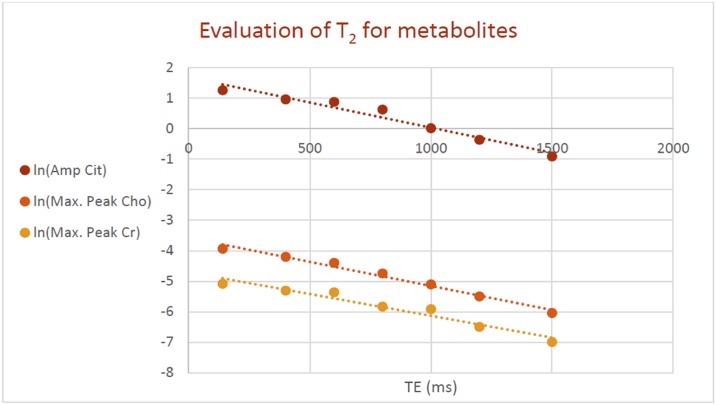
Linear regression analysis used to estimate the T_2_ parameters.

Estimation of the T_2_^m^ decay parameters was performed by acquiring phantom n°5 with PRESS sequences, using a TR of 3000 ms and echo times of 140, 400, 600, 1000, 1200 and 1500 ms, from a voxel of 2⨯2⨯2.5 cm^3^. T_2_ values of water, Cho and Cr were measured through linear regression between the logarithm of the maximum value of their peaks and TE. Since sequences based on spin-echo do not refocus the j-coupling evolution, spectrum of Cit changes dramatically varying the echo time. Consequently, T_2_ was measured by using the amplitude estimated with the homemade quantification software. In all cases, good correlations were found: r^2^ coefficients were 0.96 for Cit, 0.98 for Cho and 0.94 for Cr. T_2_^m^ measured values in phantoms were 610 ± 60 ms for Cit, 630 ± 40 ms for Cho, 700 ± 70 ms for Cr and 1220 ± 70 ms for water. Linear regression analysis is shown in Fig 7. Uncertainties on T_2_ values were estimated from linear regression analysis and were propagated on the values of the (Cho+Cr)/Cit ratios in Table 3.

## Supporting Information

S1 File “How_to_compile.txt”. Text file that contains the instructions to compile the source code “ProstateBuster.cpp” together with a list of the packages needed to install the program and the instructions to download and compile them.
(TXT)

S2 File “ProstateBuster.cpp”. This is the source code of the software.
(CPP)

S3 File “readme.txt”. Text file that contains some usage tips to run the program “ProstateBuster” and test it on the examples contained in the folders.
(TXT)

S4 File “Software_data.zip”. This is the working directory that should be copied on the user’s home folder to install and run the program (by following the instructions in “How_to_compile.txt”). It contains a copy of all the previously cited files (“How_to_compile.txt”, “ProstateBuster.cpp” and “readme.txt”) and some raw data examples to test “ProstateBuster” program.
(ZIP)

S1 Table Estimated spectral parameters of Cit inside phantoms. (DOCX)

S2 Table Estimated spectral parameters of *in vivo* Cit. (DOCX)
