# Supplementary material for: MR Spectroscopy in Prostate Cancer: New Algorithms to Optimize Metabolite Quantification
Source: PLoS One. 2016 Nov 10;11(11):e0165730. doi: 10.1371/journal.pone.0165730 (PMC5104319; doi:10.1371/journal.pone.0165730)
Supplement: S1 Table — (DOCX) [file pone.0165730.s005.docx]

**Table S1. Estimated spectral parameters of** Cit **inside phantoms.**

|  | *j (Hz)* | *Δ (ppm)* | *δ (ppm)* |
| --- | --- | --- | --- |
| pH 5 | 16 ± 2 | 0.09 ± 0.07 | 2.604 ± 0.002 |
| pH 7 | 16 ± 2 | 0.13 ± 0.05 | 2.501 ± 0.002 |
