# Supplementary material for: MR Spectroscopy in Prostate Cancer: New Algorithms to Optimize Metabolite Quantification
Source: PLoS One. 2016 Nov 10;11(11):e0165730. doi: 10.1371/journal.pone.0165730 (PMC5104319; doi:10.1371/journal.pone.0165730)
Supplement: S2 Table — (DOCX) [file pone.0165730.s006.docx]

**Table S2. Estimated spectral parameters of *in vivo*** Cit**.**

|  | *j (Hz)* | *Δ (ppm)* | *δ (ppm)* |
| --- | --- | --- | --- |
| *In vivo* | 17 ± 2 | 0.1 ± 0.5 | 2.62 ± 0.1 |
